# Emerging regulators of vascular smooth muscle cell migration

**DOI:** 10.1007/s10974-019-09531-z

**Published:** 2019-06-28

**Authors:** TecLino Afewerki, Sultan Ahmed, Derek Warren

**Affiliations:** School of Pharmacy, University of East Anglia, Norwich Research Park, Norwich, NR4 7TJ UK

**Keywords:** Vascular smooth muscle cell, Migration, Signalling, Mechanotransduction

## Abstract

Vascular smooth muscle cells (VSMCs) are the predominant cell type in the blood vessel wall and normally adopt a quiescent, contractile phenotype. VSMC migration is tightly controlled, however, disease associated changes in the soluble and insoluble environment promote VSMC migration. Classically, studies investigating VSMC migration have described the influence of soluble factors. Emerging data has highlighted the importance of insoluble factors, including extracellular matrix stiffness and porosity. In this review, we will recap on the important signalling pathways that regulate VSMC migration and reflect on the potential importance of emerging regulators of VSMC function.

## Introduction

**Fig. 1:**
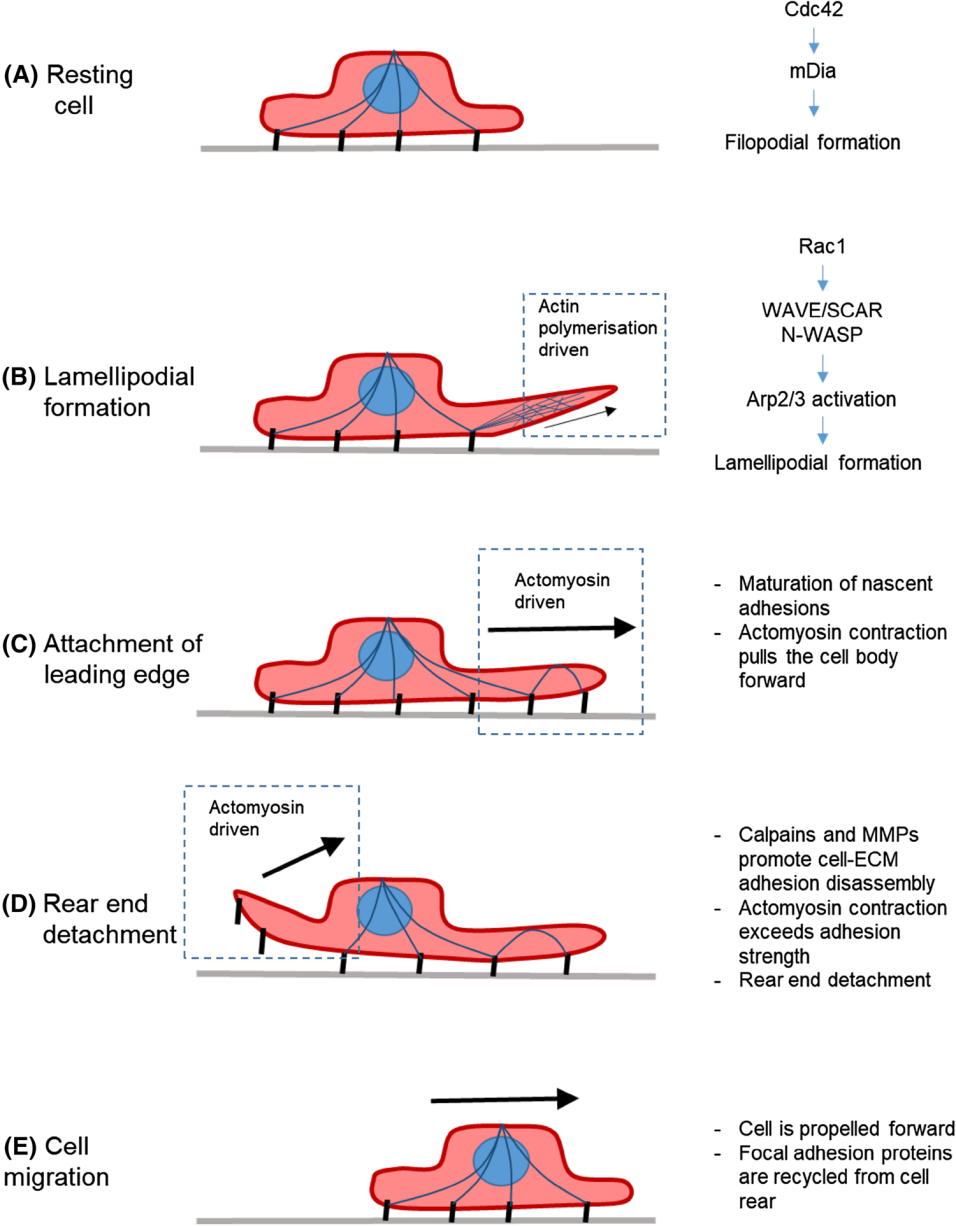
Stages of VSMC migration. Blue lines represent actin filaments. Black lines represent cell–matrix adhesions. Grey line represents extracellular matrix

Cell migration begins at the earliest stages of life and it continuous as a fundamental process for survival (Vicente-Manzanares et al. 2005). Smooth muscle cells are found in the hollow organs of the vascular, reproductive, urinary and digestive systems. In the vascular system, the vascular smooth muscle cells (VSMCs) are located in the tunica media between the loose connective tissue of the tunica adventitia and the endothelial layer of the tunica intima (Ahmed and Warren 2018). VSMC migration occurs during several important physiological and pathological processes ranging from early remodelling, response to injury, vascular disease (Tahir et al. 2015; Cai et al. 2015). VSMC migration is activated in response to multiple environmental cues including chemotactic, haptotatic and durotactic signals (Isenberg et al. 2009; Hartman et al. 2016; Kerr et al. 2013). Promigratory stimuli activate signal transduction cascades that trigger remodelling of the actin cytoskeleton and cell–extracellular matrix (ECM) adhesions that increase the migration capacity of VSMCs (Gerthoffer 2007). Similarly to other cell types, VSMC directional migration is initiated by external signals that stimulate receptors on the cell surface, activating multiple signalling cascades that alter the cytoskeletal structure of the cell (Gerthoffer 2007). Typically, stimulation is initiated via G protein coupled receptors (GPCRs) and receptor tyrosine kinases (RTKs), which in turn activate several downstream signalling pathways (Scherberich et al. 2000). Migration is a cyclical process, with external signalling inducing polarisation and filopodial projections, followed by lamellipodia extending from the cell (Louis and Zahradka 2010). This forms the leading edge of the cell. Nascent adhesions form, serving as anchor points for the newly formed protrusion to its ECM (Fig. 1). These adhesions associate with filamentous actin and actomyosin activity pulls the cell body forward (Fig. 1). Adhesions also serve as signalling conduits, allowing “inside-out” signalling by emitting the traction force generated by the cell (Ross et al. 2013). The force generated causes “outside-in” signalling to occur which further regulates the dynamics and maturation of the focal adhesions (Wrighton 2013). At the same time, adhesion disassembly and actomyosin activity detach and retract the rear of the cell, further propelling the cell body forward (Fig. 1).

Directional cell migration is often influenced by the two-dimensional and three-dimensional organization of the ECM. Recently, there have been advances in our understanding of cell migration. Specifically, around the establishment of polarity, the dynamic regulation of actin and microtubule polymerization, and the management of spatial and temporal signal transduction. We aim to address the key steps, important signalling pathways and critical regulators of aortic VSMC migration.

## Aortic mechanically properties and compliance

The medial layer of the aortic wall contains elastic and non-elastic extracellular matrix components (Wagenseil and Mecham 2009). Elastin provides the majority of the elasticity, whereas non-elastic collagen-I provides tensile strength to the medial layer (Wagenseil and Mecham 2009). This elasticity is essential for the aorta to respond to changes in blood pressure; as blood pulse moves along the vessel, the aortic wall expands and recoils behind the blood pulse returning the aorta to its original shape (Raij and Gonzalez-Ochoa 2011). This phenomenon is known as aortic compliance and this is essential for cardiovascular health (Raij and Gonzalez-Ochoa 2011). The rigidity of the aortic wall determines how compliant the vessel is; if the wall is too rigid then the blood pulse will not be sufficient to expand the aorta, too soft, then the wall expands excessively resulting in wall rupture (Karimi and Milewicz 2016; Cecelja and Chowienczyk 2012). Decreased aortic compliance is observed in multiple cardiovascular diseases, including hypertension, atherosclerosis and diabetes mellitus amongst other cardiovascular diseases (Cecelja and Chowienczyk 2012). Reduced compliance is associated with augmented arterial stiffness (Izzo and Shykoff 2001). In these conditions, the high pulse pressure is unable to expand the stiffened arterial wall, resulting in a faster pulse velocity that damages the microcirculation of vital organs (Cecelja and Chowienczyk 2012; Izzo and Shykoff 2001). Vascular smooth muscle cells are the predominant cell type in the aortic wall and disease associated changes in the mechanical landscape result in altered VSMC function (Halka et al. 2008; Shi and Chen 2015). These changes promote VSMC migration and are explored further below.

## VSMC function and phenotype

Within physiological conditions, VSMCs exist in a quiescent contractile state and use actomyosin generated force to regulate vascular tone (Brozovich et al. 2016; Galmiche et al. 2013). Intermediate filaments, microtubules and actin comprise the key filamentous components of VSMCs (Ahmed and Warren 2018). The contractile machinery of VSMCs in particular are composed of thin actin filaments and thick myosin filaments, which collectively form the actomyosin complex (Ahmed and Warren 2018). As the blood pulse travels through the aorta, VSMCs become stretched, resulting in activation of stretch activated ion channels that facilitate Ca^2+^ entry and initiate VSMC contraction (Halka et al. 2008; Berridge 2008). In addition to this mechanically regulated VSMC contraction, soluble factors, including angiotensin II, bind to receptors and activate release of Ca^2+^ from intracellular stores to promote VSMC contraction (Berridge 2008; Inagami et al. 1997). A balance between the mechanical and soluble regulation of VSMC contraction determines aortic compliance and tone, resulting in controlled organ blood flow and pressure.

Despite existing in a contractile phenotype within a mature vessel, VSMCs retain a high plasticity and can dedifferentiate into a synthetic, migratory phenotype (Owens 2007; Alexander and Owens 2012). VSMC phenotypic switching is triggered in response to changes in the mechanical/biochemical signals, which are typically associated with development and cardiovascular diseases, such as atherosclerosis and hypertension (Alexander and Owens 2012). Signals that promote VSMC phenotypic switching include growth factors, mitogens, inflammatory mediators and mechanical stimuli (Alexander and Owens 2012; Owens 2007). When dedifferentiated, VSMCs migrate from the tunica media to the tunica intima, where the cells are unable to regulate vascular tone as effectively, but gain the ability to proliferate and increase ECM synthesis (Louis and Zahradka 2010; Yu et al. 2016). Phenotypic switching is associated with a variety of changes that increase VSMC migrational capacity and these are discussed further below.

## Rho GTPases

The Rho-family of small GTPases are comprised of ~ 20 GTPases and they exist as a subfamily of the Ras superfamily (Hall 1998). They are well known to play a role in cell migration through various effectors proteins (Hall and Nobes 2000). The Rho GTPase proteins cycle between an active (GTP bound) form and an inactive (GDP bound) form. They are regulated temporally by the use of guanine nucleotide exchange factors (GEFs), GTPase-activating proteins (GAPs) and guanine nucleotide dissociation inhibitors (GDIs) (Huang et al. 2015; Bellanger et al. 2000; Ohta et al. 2006). GAPs inactivate GTPases by promoting hydrolysis of GTP into GDP, whereas GEFs prompt the exchange of GDP for GTP which triggers the enzymatic activity of the GTPase protein (Hall 1998). GDIs anchor the inactivated GTPase proteins within the cytoplasm, preventing interaction with the plasma membrane where it undergoes GDP/GTP exchange (Ridley 2001). Of these 20 GTPase proteins, RhoA, Rac1 and Cdc42 have been heavily characterised and are essential for the regulation of cell migration (Hall 1998; Hall and Nobes 2000).

The activation of RhoA is facilitated via the Gα12/13 subunit, which binds to the p115 Rho GEF and stimulates exchange of GDP/GTP (Chen et al. 2012; Patel and Karginov 2014; Siehler 2009). Once active, RhoA can activate its downstream targets, including Rho-associated protein kinase 1/2 (ROCK1/2) (Wettschureck and Offermanns 2002). ROCK1/2 are major downstream RhoA effector proteins that belong to the AGC family of serine/threonine kinases (Wettschureck and Offermanns 2002). ROCK 1 and ROCK 2 possess a large number of similarities in both structure and function. Overall, they share a 65% identity in their amino acid sequence and can be seen to modulate mutual functions in endothelial, cardiac and smooth muscle cells (Hartmann et al. 2015). However, there is evidence that there may be isoform-specific roles of these kinases (Yoneda et al. 2005; Vega et al. 2011). One example was shown in fibroblast cells, where knockdown of ROCK 1 caused aberrant adhesion maturation and actin organisation (Yoneda et al. 2005). Contrastingly, ROCK2 specifically regulates the migratory capacity of prostate cancer cells (Vega et al. 2011). ROCK1 and ROCK2 also display differential regulation of cardiomyocyte function, ROCK1 regulates apoptosis whereas ROCK2 promotes hypertrophy (Hartmann et al. 2015). Utilisation of more selective inhibitors is therefore required to identify further isoform specific roles of the ROCK1/2 kinases (Liao et al. 2007). In VSMCs, ROCK1/2 activation affects cytoskeletal remodelling in two ways; it prevents actin depolymerisation, via inhibition of cofilin, and it can increase basal levels of phosphorylated myosin light chain, by inhibiting the activity of myosin light chain phosphatase (Amano et al. 2010; Liao et al. 2007; Ridley 2001). The regulation of cytoskeletal structure and actomyosin generated force production by RhoA plays an important role in VSMC migration (Ahmed and Warren 2018). Whether ROCK1 and ROCK2 possess individual functions during VSMC migration remains to be fully explored.

Cdc42 and Rac1 also have multiple roles in cell migration. Cdc42 and Rac1 have been shown to regulate the formation of the filopodia and lamellipodia, respectively (Nobes and Hall 1995). Cdc42, via myotonic dystrophy-related Cdc42-binding kinases (MRCK), shares a similar mechanism to ROCK and induces increased myosin light chain phosphorylation (Vicente-Manzanares et al. 2005). In contrast, Lamellipodia formation via Rac1 is facilitated through Arp2/3 complex activation and the uncapping of actin filaments present at the cell membrane (Ridley 2001). In addition, both Cdc42 and Rac1 interact with and activate p21 activated kinase (PAK). Once active, PAK can increase the stabilisation of the microtubules as well as regulate cell polarity of the cell via the activation of the PIX/PAK complex (Vicente-Manzanares et al. 2005).

The GTPases are critical regulators of the migratory process and more work is required to delineate cross talk between various GTPases and to further understand their dynamic regulation during cell migration.

## Actin polymerisation

Actin polymerisation is critical for generating membrane protrusions at the leading edge during cell migration. Actin polymerisation requires nucleation via the formation of a stable actin trimer (Firat-Karalar and Welch 2011). This is the rate limiting step in the polymerisation process due to the instability of actin dimer intermediates (Firat-Karalar and Welch 2011). In order to facilitate actin polymerisation and overcome this kinetic demand, cells have evolved a wide range of actin-nucleating proteins (Firat-Karalar and Welch 2011). There are primarily two groups of actin polymerisation nucleators involved in cell migration, the formins, mDia1 and mDia2, and the Arp2/3 complex (Toure et al. 2012; Weise-Cross et al. 2015; Ning et al. 2011). mDia1 and mDia2 bind to the barbed end of actin filaments and polymerise actin linearly, to form thin cellular protrusions called filopodia (Watanabe and Higashida 2004). mDia1/2 are multi-domain proteins that function as dimers, utilising their formin homology domain 2 (FH2) to bind to globular actin monomers in order to nucleate actin (Goh and Ahmed 2012; Watanabe and Higashida 2004). The protein profilin facilitates the polymerisation via interaction with the formin homology 1 (FH1) domain of mDia1/2 (Goh and Ahmed 2012). RhoA and Cdc42 regulate the mDia1 and mDia2, respectively (Watanabe and Higashida 2004).

The Arp2/3 complex is comprised of seven proteins, two of which are Arp2 and Arp3 (Weaver et al. 2003). It promotes actin polymerisation in a branched fashion, extending actin filaments at a 70° angle from the sides of pre-existing filaments (Weaver et al. 2003). Independently, the Arp2/3 complex is intrinsically inactive, however it is activated by class I nucleation promoting factors (NPFs) (Firat-Karalar and Welch 2011). NPFs catalyse the reaction that forms lamellipodia by creating a new actin filament branch (Firat-Karalar and Welch 2011). Examples of such NPFs that regulate Arp2/3 complex activity are WAVE/Scar, Wiskott–Aldrich Syndrome protein (WASp) and N-WASp (Schenck et al. 2004; Zalevsky et al. 2001). The activity of these NPFs are primarily regulated by small GTPase proteins. WAVE/Scar is a multi-protein complex and the nucleation promoting activity is activated by Rac1, via the dissociation of the proteins Abi, Nap125 and Sra-1 from the complex (Schenck et al. 2004). In addition to this, Cdc42 has been shown to regulate both WASp and N-WASp (Vartiainen and Machesky 2004).

Cofilin is a key regulator of actin dynamics and is essential for directional cell migration. Cofilin is an actin filament severing protein and cells with low cofilin levels display defects in actin depolymerisation (Gerthoffer 2007). However, cofilin increases the dynamics of actin filament polymerisation at the leading edge by generating free barbed ends (Gungabissoon and Bamburg 2003; Ichetovkin et al. 2002). These free barbed ends promote actin filament branching via the Arp2/3 complex (Ichetovkin et al. 2002). The Arp2/3 nucleated actin branches are 10 times more stable on these recently severed free barbed ends than those produced on older filaments (Ichetovkin et al. 2002). Therefore, the functional activity of cofilin is essential in lamellipodia formation and must be tightly regulated. In resting cells, cofilin is directly bound to phosphatidylinositol 4,5-bisphosphate (PIP_2_) at the plasma membrane. Upon stimulation, cofilin is dephosphorylated to sever actin filaments and promote actin polymerisation (Van Troys et al. 2008). The actin binding protein cortactin can associate with cofilin and inhibit cofilin’s severing activity. Tyrosine phosphorylation of cortactin was found to promote cofilin dissociation from cortactin, activating the cofilin actin severing function (Van Troys et al. 2008; Gungabissoon and Bamburg 2003).

## Microtubules

Microtubules are also key modulators of VSMC migration. Cell polarisation is a prerequisite for directed cell migration, and microtubules reorient themselves into an array that faces towards the leading edge of the cell (Watanabe et al. 2005). This is achieved by selective stabilisation of microtubule plus ends and polarity is abolished by microtubule inhibitors (Watanabe et al. 2005). Dynamic instability of the microtubules is also a requirement for migration and fibroblast cell lines whose microtubules were experimentally stabilised showed a decrease in migrational capacity (Gundersen 2002). Small GTPase activity regulates microtubules dynamics. Cdc42 and Rac1 activate PAK, which phosphorylates serine 16 and subsequently inactivates stathmin, a protein that is important in microtubule destabilisation (Cassimeris 2002; Daub et al. 2001; Wittmann et al. 2004). Therefore, Cdc42 and Rac1 signalling increase the stability of microtubules during cell migration.

## VSMC–ECM adhesions

ECM adhesions are a critical component of the VSMC migratory process. During migration, VSMC–ECM adhesions assemble at the leading edge and disassemble at the trailing end (Vicente-Manzanares et al. 2005). For filopodial and lamellipodia protrusions to be maintained, they need to be physically anchored to the surrounding environment (Vicente-Manzanares et al. 2005; Ridley et al. 2003). Within VSMCs, integrins are the major receptor family that form VSMC–ECM attachments (Ross et al. 2013; Bottger et al. 1989). Integrins belong to the type I family of transmembrane glycoproteins and mediate VSMC–ECM interactions (Miyamoto et al. 1995). They are composed of three structural components; a large extracellular domain, a transmembrane domain and a short cytoplasmic domain (Miyamoto et al. 1995). Their heterodimeric (β and α subunit) isoforms allow them to have multiple compositions on the cell surface and to bind to various ECM proteins such as fibronectin and collagen-I (Seetharaman and Etienne-Manneville 2018). The beta integrin subunit links the ECM to the actin cytoskeleton (Legate and Fassler 2009; Seetharaman and Etienne-Manneville 2018). On the cytoplasmic face, adapter proteins, such as vinculin and talin, associate with the VSMC–ECM adhesion complex (Ross et al. 2013; Legate and Fassler 2009). These proteins serve to tether the integrin complex to the actin cytoskeleton. This allows for “outside in” signalling which activates signalling pathways within the cell (Wrighton 2013). Those mechanical signals cause the rearrangement of the actin cytoskeleton during migration (Ross et al. 2013). Studies conducted in rat VSMCs showed integrin to be activated by platelet derived growth factor (PDGF) which caused actin filaments to relocalise to the leading edge of the lamellipodia (Margolin et al. 2002).

Nascent VSMC–ECM adhesion formation at the leading edge of the cell requires integrin ligation, integrin clustering, phosphorylation of adhesion proteins and an intact actin filament meshwork (Miyamoto et al. 1995). Nascent VSMC–ECM adhesions are structurally small and either quickly disassemble or mature into much larger adhesions via an actomyosin dependent process (Sun et al. 2014). VSMC–ECM adhesion organisation is controlled by a wide range of kinases, including focal adhesion kinase (FAK) and integrin linked kinase (ILK) (Abedi et al. 1995; Jiang et al. 1996). Focal adhesion kinase is a tyrosine kinase composed of an N-terminal 4.1 protein, ezrin, radixin and moesin (FERM) domain, a proline-rich region and C-terminal targeting domain (Mitra et al. 2005). It has been implicated in the regulation of cell motility (Mitra et al. 2005). Phosphorylation of FAK in VSMCs has been observed in both vascular injury or when stimulated with growth factors (Abedi et al. 1995; Jiang et al. 1996; Owens et al. 2001). Crystal structure analysis reveals that FAK is maintained in an inactive conformation via molecular interaction between the FERM domain and the catalytic domain (Lietha et al. 2007). FAK functions as a scaffold protein and directly phosphorylates talin and N-WASP (Wu et al. 2004; Feller et al. 2017). Integrin linked kinase (ILK) is a serine-threonine protein kinase, with a molecular mass of ~ 50 kDa (Dedhar et al. 1999). It is an important component of the focal adhesion complex and associates with vinculin and FAK (Nikolopoulos and Turner 2001; Stanchi et al. 2009). It has been shown to activate PI3 K and inhibit glycogen synthase kinase-3β in vascular development through the modulation of downstream targets (Li et al. 2016; Dedhar et al. 1999; Ho and Bendeck 2009). However, ILK predominantly serves as a scaffold protein rather than a kinase at VSMC–ECM adhesion sites during migration (Qin and Wu 2012; Vaynberg et al. 2018). ILK is composed of three subunits; the amino N-terminal domain comprised of five ankyrin repeats, central PH-like domain and the C-terminal kinase catalytic domain (Dedhar et al. 1999). ILK co-localizes and interacts with several VSMC–ECM adhesion proteins, and such interactions coordinate actin polymerization and VSMC–ECM adhesion stability (Dedhar et al. 1999). It can also regulate the activation of Rac1 and Cdc42 by interacting with other scaffold proteins, such as α-parvin and paxillin (Stanchi et al. 2009; Nikolopoulos and Turner 2001). ILK depleted cells showed decreased wound closure due to attenuated migrational capacity (Yuan et al. 2011; Li et al. 2016).

## VSMC–VSMC adhesions

Intercellular adhesions also play an important role in migration of numerous cell types. These adhesions, much like VSMC–ECM adhesions, are structures which sense and respond to mechanical force (Borghi et al. 2012; Grashoff et al. 2010). Cadherins are the primary component of cell–cell adhesions and are comprised of three regions; an N-terminal domain, followed by a single transmembrane domain which leads to a small cytoplasmic tail (Sun et al. 2014a, b). Homodimeric interactions between the N-terminal regions of cadherin proteins from adjacent cells form the core of cell–cell contacts (Sun et al. 2014a, b). Lateral E-cadherin interactions between neighbouring cells facilitate collective migration in epithelial cells (Suffoletto et al. 2018). Within VSMCs, N-cadherin is the predominant isoform and has been shown to perform multiple roles. N-cadherin is essential for efficient VSMC migration, yet it remains unknown whether N-cadherin can facilitate collective migration of VSMCs (Lyon et al. 2010).

## Rear-end detachment

In addition to the leading edge, VSMC–ECM adhesions at the rear must detach in order to allow the cell body to propel forward (Ridley et al. 2003; Gerthoffer 2007). This is required to prevent cell damage occurring from the tension of the actomyosin generated force (Gerthoffer 2007; Vicente-Manzanares et al. 2005). Several mechanisms regulating VSMC-matrix adhesion detachment have been identified. These include contractility-promoted release, where the non-muscle myosin II contractile force exceeds the strength of the VSMC–ECM adhesion (Kirfel et al. 2004). Ca^2+^ levels also play an important role and activation of the phosphatase calcineurin, via a Ca^2+^-calmodulin dependent mechanism, also promotes adhesion detachment (Kirfel et al. 2004). Other mechanisms include modulating the affinity of the integrin for both the ECM ligands as well as the different cytosolic adaptors (Kirfel et al. 2004). Calpain and matrix metalloproteinase (MMP) activity also promote VSMC detachment (Gerthoffer 2007; Paulhe et al. 2001; Bendeck et al. 1994). Additionally, microtubules have shown to promote VSMC–ECM adhesion disassembly at the rear of the cell (Yang et al. 2010). Therefore, directed cell migration also depends on the dynamic regulation of microtubule function alongside actin polymerisation and adhesion turnover. Previous models have shown rear-end detachment plays a critical role in the reorientation and direction of the cell in migration (Theisen et al. 2012). Changes in migrational direction are higher following tail retraction, indicating that cell detachment precedes the directional change of the cell (Theisen et al. 2012). Conversely, increasing the stability of adhesions at the cell rear increased migrational directional persistence (Theisen et al. 2012).

Recently, a new mode of rapid cellular migration has been identified that involves rear end detachment to propel the cell over large distances (Wang et al. 2019). This mode is comprised of a two steps: (1) matrix stretch and (2) matrix recoil (Wang et al. 2019). Actomyosin activity first pulls ECM fibres to increase elastic strain energy (Wang et al. 2019). When this force exceeds the strength of the rear cell–ECM adhesions can withstand, it results in rapid matrix recoil and sudden cell movement via release of the strain energy (Wang et al. 2019). In addition, it was found that cell rear detachment can cause the formation of migration tracks as previous cell types, including fibroblasts and sarcoma cells, leave behind a trail of cell material which contains integrin micro aggregates (Kirfel et al. 2004; Rigort et al. 2004). This introduces a further method of rear cell detachment that could promote more efficient cell migration (Kirfel et al. 2004; Rigort et al. 2004). It is an intriguing possibility that retraction may facilitate rapid VSMC migration in 3D environments and deposit membrane fragments to promote directed migration. Whether VSMCs utilise rear end detachment to enhance their migrational capacity remains unknown and more research is required to examine the importance of retraction in VSMC migration.

## ECM composition

The ECM is comprised of various structural glycoproteins such as collagen-I, elastin, fibronectin and vitronectin (Diez 2007). In the healthy aortic wall, collagen-I and elastin are the key structural ECM proteins that determine the aortic compliance (Shirwany and Zou 2010). The ECM is remodelled during vascular ageing and disease, resulting in collagen-I accumulation and elastin degradation (Shirwany and Zou 2010). Vascular ECM remodelling is further exaggerated when VSMCs dedifferentiate into their synthetic phenotype (Diez 2007). This phenotype is associated with increased synthesis of ECM components, including collagen-I, and enhanced matrix metalloproteinase (MMPs) enzymes (Okada et al. 1992; Bendeck et al. 1994). MMPs are classified into six major subclasses based on their dependency of Zn^2+^ binding for proteolytic activity (Fanjul-Fernandez et al. 2010). They are; collagenases, gelatinases, stromelysins, matrilysins, membrane-type (MT) and other non-classified MMPs (Fanjul-Fernandez et al. 2010; Myasoedova et al. 2018). Their activity is regulated by three mechanisms which are transcription, secretion and activation of the inactive zymogens and proenzymes (Myasoedova et al. 2018; Tallant et al. 2010; Galis and Khatri 2002). The activation of secreted pro-MMPs requires proteolytic removal of the pro-domain, by disrupting the Cys-Zn^2+^ interaction (Tallant et al. 2010). Membrane type MMPS such as MT1-MMP are known to activate pro-MMPs. (Myasoedova et al. 2018) Upon activation, MMPs degrade the elastic extracellular matrix and connective tissue (Myasoedova et al. 2018; Galis and Khatri 2002). In VSMCs, tightly controlled partial degradation of the ECM is a crucial step to allow cells to reposition during remodelling. However, uncontrolled MMP activation can result in damaged ECM that enables unregulated VSMC migration (Belo et al. 2015; Galis et al. 2002).

In atherosclerotic lesions, VSMCs display altered gene expression as a result of switching to their synthetic phenotype (Owens 2007). Analysis has shown that the fibrous plaques from these lesions contain a high concentration of collagen-I/III (Raines 2000). This uncontrolled increase in collagen expression by VSMCs during migration causes the thickening of the arterial wall which ultimately results in reduced aortic compliance (Cecelja and Chowienczyk 2012). Previous research shows that there is heterogeneity in the mechanical stiffness of both healthy and diseased tissue (Tracqui et al. 2011). This heterogeneity produces stiffness gradients that promotes directional movement in VSMCs. This type of migration is known as durotaxis (Hartman et al. 2016; Isenberg et al. 2009). Within atherosclerotic plaques, VSMCs are exposed to a wide range of ECM stiffnesses, with regions ranging between 5 kPa → 200 kPa (Tracqui et al. 2011). The composition of the ECM is also important for VSMC durotaxis as fibronectin, an ECM component found in high abundance within atherosclerotic lesions, promotes VSMC directed cell migration (Hartman et al. 2016). Despite this, only a few studies have investigated how ECM composition and ECM stiffness gradients serve as VSMC directional cues.

### The nuclear lamina and VSMC migration

The nuclear envelope (NE) and nuclear lamina have surprisingly emerged as regulators of cell migration. The NE is comprised of a double lipid bilayer that forms a physical barrier between the cytoplasm and nuclear interior (Lammerding 2011). The nuclear lamina underlies the NE and provides structural support to the NE (Gruenbaum et al. 2005). The nuclear lamina is a meshwork of filamentous A-type lamins (lamins A/C), B-type lamins (lamin B1 and B2) type lamins and associated lamin binding proteins (Gruenbaum et al. 2005). Importantly, the nuclear lamina indirectly associates with cytoplasmic filamentous intermediate filaments, microtubule and actin networks. This is achieved by a NE spanning complex known as the LInker of Nucleoskeleton and Cytoskeleton (LINC) complex (Crisp et al. 2006). The LINC complex consists of giant nesprin proteins that are tethered to the outer nuclear membrane by a Klarsicht, Anc-1, Syne-1 Homology (KASH) domain (Zhang et al. 2001, 2007). The cytoplasmic facing nesprin proteins make multiple connections to the filamentous networks with multiple spectrin repeats and an N-terminal paired calponin homology domain, which directly binds filamentous actin (Zhang et al. 2001; Jayo et al. 2016; Kutscheidt et al. 2014). LINC complex stability is maintained via interactions between the nesprin KASH domain and the SUN-domain of the SUN family of proteins in the perinuclear space (Crisp et al. 2006; Haque et al. 2006; Sosa et al. 2012). The SUN proteins span the inner nuclear membrane and interact with lamins A/C in the nucleoplasm (Haque et al. 2010). Cytoskeletal derived biophysical signals are transmitted across the LINC complex and regulate association of the nuclear lamina with the NE and activated nuclear signalling (Arsenovic et al. 2016; Guilluy et al. 2014; Wu et al. 2014). The LINC complex also transmits inside out signals from the nuclear lamina to the cytoskeleton as disruption of the LINC complex or nuclear lamina triggers cytoskeletal reorganisation, altered GTPase signalling and impaired cell migration (Schwartz et al. 2017; Chambliss et al. 2013). However, the nature of these inside out signals remains unknown.

The A-type lamins are encoded by the LMNA gene (Rusinol and Sinensky 2006). Lamin C is directly generated from the LMNA gene (Rusinol and Sinensky 2006). In contrast, lamin A is generated from the prelamin A precursor protein that requires several post translational modification steps to generate mature lamin A (Rusinol and Sinensky 2006). Prelamin A is farnesylated and is inserted into the INM (Rusinol and Sinensky 2006). To release mature lamin A from the INM, prelamin A must be processed by the metalloproteinase FACE1, which cleaves the C-terminal 15 amino acids of prelamin A, that include the farnesyl modification, to yield soluble mature lamin A (Rusinol and Sinensky 2006; Corrigan et al. 2005). Improper prelamin A processing is observed in Hutchinson–Gilford progeria syndrome (HGPS) accelerated ageing and in normal VSMC ageing. HGPS patients display accelerated ageing and possess a mutation that removes the FACE1 cleavage site in prelamin A (Rusinol and Sinensky 2006). This results in defective lamin A processing and prelamin A accumulation at the INM. VSMCs are severely affected in HGPS, suggesting that VSMCs are extremely sensitive to changes in lamin A processing (Ragnauth et al. 2010; Olive et al. 2010; McClintock et al. 2006). Prelamin A accumulation is also observed in normal VSMC ageing and is driven by loss of FACE1 expression (Ragnauth et al. 2010). In both situations, prelamin A accumulation is proposed to interfere with mature lamin A function. HGPS-derived fibroblast cells exhibit cytoskeletal reorganisation, cell–ECM adhesion remodelling and reduced migrational capacity (Chang et al. 2019; Booth-Gauthier et al. 2013). Importantly, prelamin A accumulation or lamin A depletion in VSMCs showed similar changes in cytoskeletal and cell–ECM adhesion organisation as HGPS-derived fibroblasts (Porter et al. 2016). In VSMCs, the nuclear lamina influences migrational directional persistence via regulation of Rac1 (Porter et al. 2016). Although the mechanism of this regulation remains unknown, it is likely that the LINC complex is involved. Further experimentation is required to better understand the role of the nuclear lamina and LINC complex in VSMC migration within physiological and pathological environments.

### DNA damage and 3D migration

**Fig. 2:**
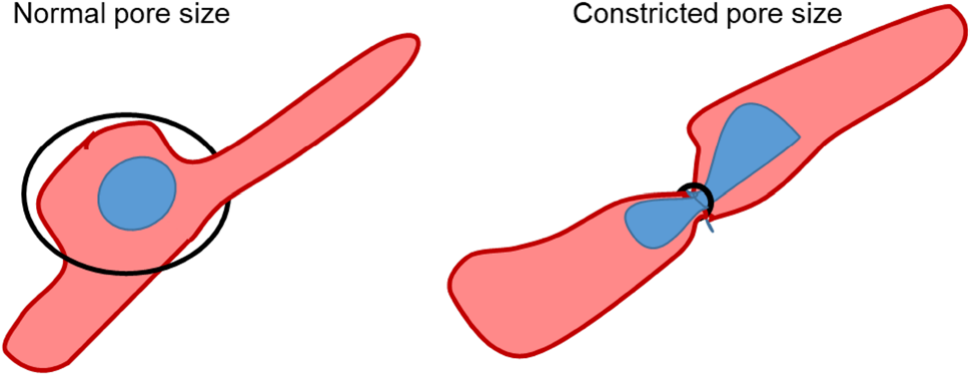
Confined VSMC migration

Much of our knowledge discussed above has been generated on 2D surfaces. In vivo, cells must navigate complex 3D environments. The ECM is a porous network and as VSMCs move, they secrete MMPs to expand these pores, so VSMC migration is unhindered (Wolf et al. 2013). However, vascular calcification, where blood vessel walls calcify and become similar to bone, is observed in many vascular diseases, including atherosclerosis and diabetes (Liu and Shanahan 2011). In these conditions, VSMCs cannot degrade the calcified ECM to expand the pore size. How VSMCs migrate in these conditions remains completely unknown. Other cell types undergo a process known as constricted migration under conditions where they are unable to increase pore size (Wolf et al. 2013). The cytoplasm is not limited by ECM pore size, however, the nucleus is the largest and stiffest cellular organelle (Wolf et al. 2013) (Fig. 2). For cells to migrate though pores of limited size, cells use actomyosin generated force to squeeze the nucleus though (Davidson et al. 2014) (Fig. 2). This process results in nuclear deformation and places considerable pressure on the NE that ultimately results in NE damage and rupture (Denais et al. 2016; Xia et al. 2018). The nuclear envelope is a protective barrier separating the nuclear content from the cytoplasm. This allows compartmentalisation of crucial molecular components which facilitate processes such as DNA replication and RNA synthesis. NE rupture results in loss of this barrier function and induces DNA damage, which is predicted to promote genomic mutations that accelerate cancer metastasis (Xia et al. 2018; Irianto et al. 2017; Denais et al. 2016). DNA damage accumulation is known to accelerate prelamin A accumulation and VSMC ageing (Ragnauth et al. 2010). Aged VSMCs adopt a secretory phenotype that further accelerates blood vessel calcification (Liu et al. 2013). Therefore, VSMCs undergoing confined migration may promote the secretory phenotype and further accelerate vascular ageing (Liu et al. 2013; Ragnauth et al. 2010). Whether VSMCs undergo constricted migration within the calcified vessel wall remains unknown. However, given the importance of DNA damage in driving VSMC dysfunction and vessel wall deterioration, this remains a key unanswered question.

### Future directions

Much research exists into the role of soluble factors and biochemical signalling in VSMC migration. However, we lack an understanding of two key areas: (1) how biophysical signalling and intercellular coupling between the cytoskeleton and nucleus contributes to VSMC migration; and (2) the role of insoluble factors, including matrix stiffness and porosity. Biophysical signalling and insoluble cues are known to change during ageing and vascular disease. Therefore, unravelling the mechanistic role of these changes will potentially identify novel therapeutic pathways to manipulate VSMC migration in ageing and vascular disease.
